# Volanesorsen: A New Era in the Treatment of Severe Hypertriglyceridemia

**DOI:** 10.3390/jcm11040982

**Published:** 2022-02-13

**Authors:** Genovefa Kolovou, Vana Kolovou, Niki Katsiki

**Affiliations:** 1 Metropolitan Hospital, Cardiometabolic Center, Lipoprotein Apheresis and Lipid Disorders Clinic, 9 Ethn. Makariou & 1 El Venizelou Str., N Faliro, 185 47 Athens, Greece; bkolovou@gmail.com; 2 First Department of Internal Medicine, Diabetes Center, Division of Endocrinology and Metabolism, AHEPA University Hospital, 541 24 Thessaloniki, Greece; nikikatsiki@hotmail.com

**Keywords:** familial chylomicronemia syndrome, volanesorsen, lipoprotein lipase mutation, hypertriglyceridemia, pancreatitis

## Abstract

Introduction: Familial chylomicronemia syndrome (FCS) is a rare inherited disease, mainly due to lipoprotein lipase (*LPL*) gene mutations, leading to lipid abnormalities. Volanesorsen, a second-generation 2′-*O*-methoxyethyl (2′-MOE) chimeric antisense therapeutic oligonucleotide, can decrease plasma apolipoprotein C3 and triglycerides (TG) levels through LPL-independent pathways. The European Medicines Agency has approved volanesorsen as an adjunct to diet in adult FCS patients with an inadequate response to TG-lowering therapy. Areas covered: Available clinical data on volanesorsen efficacy and safety are presented. Furthermore, we discuss the yearly treatment with volanesorsen of a 21-year-old female FCS patient with *LPL* mutation. Volanesorsen was well-tolerated and decreased patient’s TG levels (from >5000 mg/dL (56 mmol/L) to 350–500 mg/dL (4–5.6 mmol/L)) at 12 months. Lipoprotein apheresis (LA) was stopped and there were no episodes of pancreatitis or abdominal pain. Expert opinion: Severe hypertriglyceridemia can potentially be fatal. Until recently, there was no specific treatment for FCS, apart from hypotriglyceridemic diet, fibrates, omega-3 fatty acids, and LA sessions. Therefore, volanesorsen represents a promising therapeutic solution for these patients. The main side effect of volanesorsen therapy is thrombocytopenia, which should be monitored and treated accordingly. Increasing evidence will further elucidate the clinical implications of volanesorsen use in daily practice.

Hypertriglyceridemia (mild/moderate, TG: 150–499 mg/dL (1.7–5.6 mmol/L); severe, TG: >500 mg/dL (5.6 mmol/L); extreme, TG: >2000 mg/dL (22 mmol/L)) can potentially be a fatal condition, particularly in primary cases such as in familial chylomicronemia syndrome (FCS).

FCS is a rare disease (1:1,000,000 for homozygote and 1 in 500 for heterozygote) inherited in an autosomal recessive manner (genetic causes of hypertriglyceridemia are grouped under FCS) and is mainly due to lipoprotein lipase (*LPL*) gene mutations. 

Volanesorsen, a second-generation 2′-*O*-methoxyethyl (2′-MOE) chimeric antisense therapeutic oligonucleotide (ASO), has been approved as an adjunct to diet in adult FCS patients with an inadequate response to TG-lowering treatment. 

Volanesorsen efficacy and safety in FCS has been proven in several studies, including randomized, double-blind, placebo-controlled studies and meta-analyses.

The main side effect of volanesorsen use is thrombocytopenia. Therefore, in patients treated with volanesorsen, platelet count should be monitored at a weekly basis and kept >140,000/μL.

## 1. Introduction

Hypertriglyceridemia is defined as mild/moderate (plasma triglyceride (TG): 150–499 mg/dL (1.7–5.6 mmol/L)), severe (plasma TG: >500 mg/dL (5.6 mmol/L)), or extreme (>2000 mg/dL (22 mmol/L)) and can be fatal, as shown in a series of 221 patients with TG-induced pancreatitis [1]. Patients with hypertriglyceridemia may also present with emotional, cognitive and psychosocial symptoms, thus reducing their quality of life and limiting their employment and social interactions as documented by Davidson al, in a study of 166 patients with familial chylomicronemia syndrome (FCS) [2]. Furthermore, hypertriglyceridemia can have primary or secondary causes (e.g., high-fat diet, excessive alcohol consumption, obesity, uncontrolled diabetes mellitus, hypothyroidism, renal or liver disease, autoimmune disorders, pregnancy, and use of medications such as beta blockers, thiazides, estrogen, corticosteroids, antipsychotics, bile acid-binding resins, immunosuppressants and antiretroviral protease inhibitors) [3]. FCS is a rare disease (1:1,000,000) inherited in an autosomal recessive manner and is mainly due to lipoprotein lipase (*LPL*) gene mutations, although other genes can also be involved such as encoding proteins required for LPL activity, apolipoprotein (Apo) C2, ApoA5, lipase maturation factor-1 (LMF-1), and glycosylphosphatidylinositol-anchored high-density lipoprotein–binding protein 1 (GPIHBP1) [4]. All these mutations can lead to lipid abnormalities. LPL is required for the hydrolysis of TG in chylomicrons (CMs) and very-low-density lipoprotein (VLDL) particles. In patients with FCS, fasting plasma has a milky, white appearance, and, if left undisturbed for some hours, a creamy layer of CMs can be clearly observed on the top [5]. Fasting TG levels are usually >1000 mg/dL (11 mmol/L) and can even reach >10,000 mg/dL (110 mmol/L) [6]. Elevations of total cholesterol concentrations can also occur, but to a lesser degree. 

FCS can be diagnosed in childhood or early adulthood due to the accumulation of CMs in the plasma, causing eruptive xanthomas, lipemia retinalis (visible intraarterial hypertriglyceridemia), recurrent incidents of abdominal pain and/or pancreatitis, milky white plasma (large increase in plasma CM levels) and TG levels > 1000 mg/dL (11 mmol/L). 

Volanesorsen was approved by the European Medicines Agency (EMA) in 2019 (https://www.ema.europa.eu/en/documents/product-information/waylivra-epar-product-information_en.pdf accessed on 16 December 2021) as an adjunct to diet in adult patients with FCS in whom response to diet and TG-lowering therapy have been inadequate. Volanesorsen is a second-generation 2′-*O*-methoxyethyl (2′-MOE) chimeric antisense therapeutic oligonucleotide (ASO) that selectively reduces apolipoprotein (apo)C3 messenger ribonucleic acid (mRNA), leading to dose-dependent decreases in plasma apoC3 and TG levels through LPL-independent pathways [7].

## 2. Body

### 2.1. Case Presentation

G.N. is a 21-year-old woman with FCS treated with volanesorsen. At the age of 2 years, she was hospitalized for acute pancreatitis with elevated TGs > 4000 mg/dL (45 mmol/L). She was then clinically diagnosed with primary chylomicronemia and treated with a special diet devoid of TG and containing medium-chain fatty acids; she was also started on fenofibrate 145 mg daily. Until the age of 16 years, she was hospitalized for acute/subacute pancreatitis several times (6–9 episodes per year) and her plasma TG levels were between 2000 and 7000 mg/dL (22.6 and 79 mmol/L). 

At her first visit to our lipid clinic, physical examination did not reveal any pathological findings, i.e., the abdomen was soft and non-tender and there were no eruptive xanthomas or dysmorphic features. Blood was drawn, revealing the characteristic milky white appearance. The patient’s TG levels were >5000 mg/dL (56 mmol/L), whereas all other biochemical tests were within the normal range. The patient’s family history was unremarkable. Genetic analysis revealed the presence of an *LPL* mutation (*Gly215Glu/Met328Arg*). 

She was advised to continue the special TG-lowering diet, consuming medium-chain fatty acids, switch fenofibrate to gemfibrozil 1.800 mg/day, and take 6 g of omega-3 fatty acids daily. Additionally, she was treated regularly (every week) with LA. With this treatment, her plasma TG levels were maintained between 1200 and 3000 mg/dL (13 and 34 mmol/L). Unfortunately, the episodes of pancreatitis continued, although less frequently (i.e., 3–4 episodes per year). In 2019, volanesorsen (an apoC3 inhibitor) was approved for the treatment of FCS in Europe. Therefore, in early March 2020, we added volanesorsen 285 mg/weekly to gemfibrozil 1800 mg/day and 6 g omega-3 fatty acids/day. LA was stopped when volanesorsen was initiated. After one year of treatment with volanesorsen, her TG levels dropped down to 350–500 mg/dL (4–5.6 mmol/L) with no episode of pancreatitis or abdominal pain. Volanesorsen was well-tolerated. Of note, during this one year of volanesorsen use, she reported not taking gemfibrozil regularly, as well as stopping omega-3 fatty acids intake and drinking alcohol beverages sometimes; in such cases, her TG levels increased to 500–800 mg/dL (5.6–9 mmol/L) with no abdominal pain or pancreatitis occurrence. 

Following the initiation of volanesorsen therapy, her platelet count was checked every week. In the fifth week, platelets were decreased to <100,000/μL and volanesorsen was stopped for 2 weeks. After patient’s platelets returned to >140,000/μL, volanesorsen was restarted until now without any further side effects. We are still checking her platelet numbers weekly. The patient self-reported that this treatment improved her quality of life, since she is not traveling (200 km) for LA treatment and has had no hospital admission for acute pancreatitis. 

### 2.2. Clinical Data on Volanesorsen Efficacy and Safety 

Volanesorsen efficacy and safety in FCS were initially evaluated in an open-label, 13-week clinical trial including three patients with FCS and TG levels ranging from 1406 to 2083 mg/dL (15.9 to 23.5 mmol/L) [8]. Plasma apoC3 and TG levels were reduced from baseline by 71% to 90% and 56% to 86%, respectively. During the study, all patients had a triglyceride level of less than 500 mg/dL (5.7 mmol/L) with treatment. There were also marked reductions in cholesterol concentration in CMs and VLDL particles (51% to 83% from baseline), as well as in non-HDL-C (46% to 74% from baseline) [8].

In another single-center, 13-week, phase 2, randomized, double-blind, placebo-controlled study, involving 57 untreated patients with fasting TGs between 350 and 2000 mg/dL (4.0 and 22.6 mmol/L, respectively) and 28 patients on stable fibrate therapy with fasting TGs between 225 and 2000 mg/dL (2.5 and 22.6 mmol/L, respectively), volanesorsen led to dose-dependent and prolonged decreases in plasma apoC3 and TG concentrations in the monotherapy and add-on to fibrate group [9]. Of note, volanesorsen was administered at 100, 200, and 300 mg/week in monotherapy groups and at 200 and 300 mg/week in the fibrate groups. In brief, plasma apoC3 was significantly reduced by 40%, 63.8%, and 79.6% in the 100, 200, and 300 mg volanesorsen monotherapy groups, respectively, and by 60.2% and 70.9% in the 200 and 300 mg volanesorsen + fibrate groups, respectively; corresponding values for TG levels were 31.3%, 57.7%, and 70.9% for monotherapy groups and 51% and 64% for the volanesorsen + fibrate groups, respectively [9]. Dose-dependent reductions in VLDL were also observed with volanesorsen (monotherapy + combination therapy). Of note, LDL-C was dose-dependently increased with volanesorsen, but only in the monotherapy groups (and not in the fibrate groups), from a mean baseline level of 80 ± 30 mg/dL (2.1 ± 0.8 mmol/L) to a mean level of 128 ± 45 mg/dL (3.3 ± 1.2 mmol/L) at the end of treatment [9]. However, this increase in LDL-C level was accompanied by an increase in LDL particle size, thus potentially minimizing CV risk [10,11]. Due to the close association between the most atherogenic, small, dense LDL (sdLDL) and TGs, it follows that TG-lowering can lead to sdLDL reductions [12]. In terms of safety issues, local reactions at the injection site occurred in 13% and 15% of injections in the monotherapy and volanesorsen + fibrate cohorts, respectively; these reactions were typically mild and resolved spontaneously. Overall, 2/20 volanesorsen + fibrate treated patients (10%) presented with diarrhea, 2/20 (10%) with abdominal pain, 2/20 (10%) with fatigue, and 2/20 (10%) with feelings of relaxation. In the volanesorsen monotherapy groups, 6/41 patients (14.6%) mentioned fatigue, 4/41 (9.8%) musculoskeletal pain, 4/41 (9.8%) nausea, 3/41 (7.3%) chills, and 3/41 (7.3%) myalgia [9]. In the placebo cohorts, only 1/16 (6.3%) patients in monotherapy reported fatigue and 1/8 (12.5%) in the fibrate group had diarrhea. Of patients treated with volanesorsen, 6/61 (10%) discontinued the drug due to adverse events. Furthermore, there was no evidence (clinical or laboratory) of drug–drug interactions in patients on concomitant medications (statins, fibrates, glucose-lowering agents, etc.) [9].

In another single-center, randomized, double-blind, placebo-controlled trial, including 15 patients with type 2 diabetes (HbA1c > 7.5% (58 mmol/mol)) and hypertriglyceridemia (TG > 200 and <500 mg/dL; >2.3 and <5.6 mmol/L), patients were randomized in a 2:1 ratio to receive volanesorsen 300 mg or placebo for 15 subcutaneous weekly doses [13]. Volanesorsen significantly lowered plasma apoC3 and TG levels (by 88% and 69%, respectively; *p*-values 0.02 for both comparisons) and increased HDL-C concentrations (by 42%; *p* = 0.03) compared to the placebo. The reductions in TG and apoC3 strongly correlated with improvements in insulin sensitivity, and HbA1c was significantly decreased by 0.44%, even at 3 months postdosing [13]. The most frequent adverse events were local cutaneous reactions at the injection site (in 15% of all injections), followed by headache (in five patients) and upper respiratory tract infection (in five patients). The majority of these side effects were mild and there were no clinically relevant changes in serum or urine biomarkers, electrocardiogram, or vital signs [13]. 

A more recent (2019) multicenter, phase 3, double-blind, randomized 52-week trial (the APPROACH trial) evaluated the effectiveness and safety of volanesorsen in 66 patients with FCS [14]. Patients were randomly assigned to receive either volanesorsen at a dose of 300 mg subcutaneously once a week or placebo. At 3 months, apoC3 levels were significantly reduced (by 84%) in the volanesorsen-treated patients, whereas they increased in the placebo group (by 6.1%; *p* < 0.001). Similarly, volanesorsen therapy significantly decreased TG levels (by 77%, corresponding to a mean decrease of 1712 mg/dL (19.3 mmol/L)), whereas they were elevated in the placebo group (by 18%, corresponding to a mean increase of 92.0 mg/dL (1.0 mmol/L); *p* < 0.001) [14]. Volanesorsen’s beneficial effects were sustained for 6 months for both apoC3 and TG reductions (by a mean of 83% and 53%, respectively). For TGs, the between-group difference in the percentage change was −77.8% (95% confidence interval (CI): −106.4 to −49.1; *p* < 0.001). At 12 months, TG levels were lowered by 40% in the volanesorsen group and increased by 9% in the placebo group (between-group relative difference in percentage change: −49.1% (95% CI: −94.7 to −3.5; *p* = 0.03)) [14]. Overall, 77% of the volanesorsen-treated patients (and only 10% of patients in the placebo group) had TGs < 750 mg/dL (8.5 mmol/L). The TG-reducing effect of volanesorsen was irrespective of the patients’ genetic diagnoses or concomitant TG-lowering treatment with omega-3 fatty acids, fibrates, or both. Furthermore, the levels of apo B-48 (by 76%), non-HDL-C (by 46%), and VLDL (by 58%) decreased, and HDL-C (by 46%), apoA1 (by 14%), apoB (by 20%), and LDL-C (by 136%) levels increased [14]. In exploratory analyses, adjudicated episodes of acute pancreatitis were assessed during the trial; three patients in the placebo group had four episodes of acute pancreatitis, whereas only one patient in the volanesorsen group had one episode (and this occurred 9 days after the final dose administration).

With regard to safety, 20/33 patients (61%) who received volanesorsen had injection-site reactions and 16/33 (48%) had platelet counts <100,000/μL (two patients had even <25,000/μL and were discontinued from the trial); such adverse events were not observed in the placebo group [14]. After the occurrence of the two cases of severe thrombocytopenia, an intensive platelet monitoring program was implemented consisting of platelet count assessment every 2 weeks. Dose frequency was to be reduced to every 2 weeks if platelets were lowered <100,000/μL and dosing was to be interrupted at the level of 75,000/μL. Following these rules, no patient had platelet count declines <50,000/μL and no platelet-related dose discontinuations were observed [14]. Apart from platelet count reduction, the other side effects reported in ≥10% of volanesorsen-treated patients were abdominal pain (27%), fatigue (21%), headache (21%), nausea (18%), myalgia (15%), diarrhea (15%), epistaxis (15%), vomiting (15%), nasopharyngitis (15%), petechiae (12%), arthralgia (12%), and diabetes (12%). Abdominal pain (21%), fatigue (9%), headache (15%), nausea (6%), myalgia (3%), diarrhea (6%), vomiting (9%), and nasopharyngitis (21%), were also recorded in the placebo group [14]. Among volanesorsen-treated patients, nine discontinued the drug due to adverse events (five due to platelet count decreases and four due to other volanesorsen-related effects, i.e., injection-site reaction, fatigue, chills, sweating, and generalized erythema) [14].

Very recently (30 March 2021), the results of the COMPASS trial, a multicenter, phase 3, randomized, double-blind, placebo-controlled study, involving 114 patients with multifactorial severe hypertriglyceridemia or FCS (baseline TGs ≥ 500 mg/dL (5.6 mmol/L)), were published online [15]. Patients received either volanesorsen 300 mg (*n* = 76) or placebo (*n* = 38) for 13 weeks. At 3 months, volanesorsen significantly decreased mean plasma TGs by 71.2% (95% CI: −79.3 to −63.2); TG levels increased by 0.9% (−13.9 to 12.2) in the placebo group (*p* < 0.0001). These results represent a mean absolute lowering of TGs by 869 mg/dL in volanesorsen-treated patients compared to an increase of 74 mg/dL in the placebo [15]. During the study treatment period, five adjudicated events of acute pancreatitis were recorded in 3/38 patients in the placebo group. The most frequent side effects were mild-to-moderate injection-site reactions (in 24% and 0.2% of all volanesorsen and placebo injections, respectively) [15]. Serious adverse events were reported in two volanesorsen-treated patients and involved one case of platelet count reduction to <50,000/μL and one case of serum sickness. Overall, these findings support a role for volanesorsen in reducing TGs and acute pancreatitis events in patients with FCS or severe hypertriglyceridemia. 

A previous (2020) meta-analysis of three randomized controlled clinical trials (*n* = 156 subjects) found that volanesorsen significantly decreased TG (by 68%), VLDL (by 73%), apoB48 (by 65%), and apoC3 (by 75%) levels, as well as increased HDL-C levels (by 40%) [16]. These effects on lipids may beneficially affect cardiovascular risk, as elevated TG and apoC3 levels can predispose patients to atherosclerosis and coronary heart disease [17,18,19]. Of note, omega-3 fatty acids have also been shown to decrease TG and apoC3 levels [20,21]. 

The Retrospective Findings and Observations Captured in Burden of Illness Survey in FCS Patients (ReFOCUS) study was a retrospective, global, web-based survey for patients with FCS who received volanesorsen for at least 3 months [22]. Among 22 participants who received volanesorsen therapy for a median of 222 days, an overall improved management of symptoms and decreased impact of FCS on work/school responsibilities and social and personal life was reported. The frequency of pancreatic pain and steatorrhea was also significantly reduced and patients worried less about an attack of pain or acute pancreatitis [22]. Furthermore, responders reported an easier dietary management of their disease during volanesorsen treatment. These findings highlight the efficacy of volanesorsen in decreasing disease burden in FCS patients. 

## 3. Conclusions

Volanesorsen can be considered in the treatment of patients with severe hypertriglyceridemia or FCS to lower their elevated TG and apoC3 levels, prevent acute pancreatitis recurrence and improve their symptoms, thus facilitating their professional, social, and personal life. EMA has approved volanesorsen as an adjunct to diet in adult patients with FCS who have an inadequate response to TG-lowering therapy. Safety issues have been raised in relation to volanesorsen therapy, especially involving platelets, and thus a platelet count monitoring program should be implemented in all volanesorsen-treated patients, until new safety data are available. Furthermore, whether volanesorsen-induced TG and apoC3 reductions will translate to cardiovascular prevention remains to be established. 

## 4. Expert Opinion

The major health problem in patients with FCS is the recurrence of acute pancreatitis episodes, requiring several days of hospitalization, even in the intensive care unit, and potentially leading to death. Diabetes mellitus may also be developed as a complication of chylomicronemia. Pancreatic damage is caused by increased free fatty acids levels, leading to the activation of trypsinogen (a digestive enzyme, the precursor of trypsin) [23]. Furthermore, CMs may impair distal pancreatic circulation and induce ischemia, thus resulting in acinar cells dysfunction and exposure of the pancreatic tissue to TGs [23,24]. Of note, acinar cells synthesize and secrete almost all the digestive enzymes that are active in the lumen of the small intestine and that are necessary for nutrient digestion. Finally, pancreatic lipase is activated, leading to autoinflammation (i.e., damage to host tissues due to the dysregulated secretion of pro-inflammatory cytokines by activated innate immune cells [25]). 

In patients with severe/extreme hypertriglyceridemia, such as those with FCS, the primary therapeutic target is to lower TG levels and thus prevent episodes of acute pancreatitis. Until recently, there was no specific treatment for FCS, apart from hypotriglyceridemic diet, fibrates, omega-3 fatty acids, and LA sessions [26]. LA treatment of patients with severe hypertriglyceridemia may significantly reduce their TG levels. For example, a multicenter study of 17 patients with severe hypertriglyceridemia non-responding to conventional medical therapy, reported by Stefanutti et al. [27], showed that the removal of TG-rich lipoproteins by plasmapheresis prevented relapses of acute pancreatitis and proved to be a safe and reliable therapeutic method. Similarly, Ewald and Kloer [28], in their review, reported that a single session of plasmapheresis lowered TG concentrations by up to 70% and provided significant improvements in clinical symptoms as well as in morbidity and mortality. However, LA treatment is available only in selected centers and several patients must travel hundreds of kilometers to reach the nearest center. Furthermore, LA therapy may be associated with adverse events (most likely during the first sessions) such as allergic reactions manifested as shortness of breath and/or facial flushing, nausea, vomiting, and serious hypotension; other limitations include venipuncture difficulties and technical problems [29,30]. All the above can further hamper a patient’s quality of life [30].

Volanesorsen promotes TG clearance and the lowering of plasma TG levels through LPL-independent pathways [7]. Therefore, volanesorsen treatment was shown to be related to dose-dependent decreases in plasma apoC3 and TG levels [31]. Based on these effects and the positive results from phase 2 and 3 clinical studies, supporting its efficacy in patients with severe hypertriglyceridemia or FCS, volanesorsen was approved by the EMA for use in adult patients with FCS who have an inadequate response to diet and TG-lowering therapy. Volanesorsen was shown to reduce TG levels (by 30–70%), improve patients’ well-being, and decrease or prevent episodes of pancreatitis.

In terms of safety issues, the most frequent side effects of volanesorsen were mild-to-moderate injection-site reactions, followed by headache, fatigue, symptoms from the gastrointestinal tract (e.g., abdominal pain, nausea, diarrhea, vomiting), myalgia/arthralgia, and nasopharyngitis. The majority of these adverse events were mild in severity and did not lead to clinically relevant changes in urine or serum biomarkers, electrocardiogram, or vital signs. A potentially serious side effect reported in volanesorsen-treated patients is thrombocytopenia; thus, platelet count should be frequently monitored.

All women on hypolipidemic treatment, including volanesorsen, should be advised to have programmed pregnancy and to stop hypolipidemic treatment at least one month before pregnancy. In pregnant women, acute pancreatitis is uncommon, with a prevalence rate of 1/1000–12,000 pregnancies, but it has been associated with an increased frequency of an abnormal maternal outcome [32]. If fasting TG levels become very high during pregnancy, lipoprotein apheresis sections should be performed [33]. Therapeutic plasmapheresis can lead to a rapid decrease in TG concentrations, thus preventing complications induced by severe episodes of hypertriglyceridemia [34,35]. In this context, Basar et al. reported that apheresis effectively reduced TG levels in pregnant women with severe hypertriglyceridemia [36]. Similar results were found in another study involving women with hypertriglyceridemia-induced acute pancreatitis during pregnancy [37]. In this study, plasmapheresis decreased TG levels, the length of hospitalization and prevented the incidence of systemic inflammatory response syndrome [37]. Overall, plasma exchange (apheresis/plasmapheresis) represents an effective and safe treatment option for severe gestational hypertriglyceridemia.

Overall, despite its high cost, subcutaneous administration and possibility of causing thrombocytopenia, volanesorsen may be useful in clinical practice in treating patients with FCS (and severe hypertriglyceridemia) when other TG-lowering therapies are proven inadequate to control TG levels and prevent pancreatitis. Further research in apoC3 inhibition will establish whether FCS can become a treatable disorder.

## Data Availability

Data supporting reported results are stored in Metropolitan Hospital’s archives and can be sent after contacting the authors.
